# Editorial: Anti-cancer effects of botanical drugs targeting the tumor microenvironment

**DOI:** 10.3389/fphar.2023.1268094

**Published:** 2023-08-17

**Authors:** Chien-shan Cheng, Ning Wang, Zhiqiang Meng

**Affiliations:** ^1^ Ruijin Hospital, School of Medicine, Shanghai Jiao Tong University, Shanghai, China; ^2^ School of Chinese Medicine, LKS Faculty of Medicine, The University of Hong Kong, Pok Fu Lam, Hong Kong SAR, China; ^3^ Department of Integrative Oncology, Fudan University Shanghai Cancer Center, Shanghai, China

**Keywords:** botanical drug, natural product, tumor micreoenvironment (TME), therapeutic advance, anti-cancer (anticancer) drugs

It is with great pleasure and enthusiasm that we present this Research Topic, “*Anti-cancer Effects of Botanical Drugs Targeting the Tumor Microenvironment*” This Research Topic of groundbreaking research delves into an exciting and promising area of cancer research, where the tumor microenvironment takes center stage as a critical factor influencing cancer development, progression, and therapeutic responses. The diverse range of articles featured in this Research Topic sheds light on the innovative use of botanical drugs to modulate the tumor microenvironment and unlock their potential as effective anti-cancer agents through the modulation of the tumor microenvironment. We extend our heartfelt appreciation to the authors, reviewers, and the dedicated editorial team for their unwavering commitment to advancing knowledge in this vital field.

Over the years, the understanding of cancer biology has evolved, leading to a paradigm shift in cancer treatment strategies. Targeted therapies and immunotherapies have shown significant success, but challenges such as resistance and side effects persist. It is in this context that the role of botanical drugs comes into focus. With their diverse array of bioactive compounds and complex interactions, botanical drugs offer unique opportunities for precise and holistic cancer treatments. This Research Topic received significant interest, resulting in an impressive 47 manuscript submissions. After a rigorous peer-review process, 14 of these manuscripts were deemed suitable for publication, showcasing the high quality and relevance of the selected works. The studies featured in this Research Topic shed light on the untapped potential of botanical drugs in targeting cancer cells and their surrounding microenvironment.

One of the key aspects explored in this Research Topic is the concept of precision medicine. As we delve deeper into the genomic and molecular heterogeneity of cancer, the need for personalized treatment approaches becomes evident. A significant contribution comes from the study contributed by Wei et al. in which, through intricate analysis of gene signatures, this research advances our ability to predict patient outcomes and stratify osteosarcoma subtypes, paving the way for personalized treatment approaches. Intriguingly, the integration of risk stratification systems and machine learning algorithms has enabled the identification of gene signatures associated with poor overall survival and treatment resistance. Moreover, the exploration of pyroptosis-related genes and their correlation with patient outcomes and tumor-infiltrating cells by Xie et al. offers new insights into the potential of targeting pyroptosis for therapeutic intervention and contributes to our understanding of cancer heterogeneity and personalized treatment approaches.

Several articles in this Research Topic emphasize the potential of botanical drugs in identifying and targeting specific biomarkers within the tumor microenvironment. Among the remarkable contributions in this Research Topic contributed by Li et al. highlights the potential of botanical extracts in enhancing the efficacy of existing chemotherapeutic agents, offering new hope in overcoming drug resistance. In the realm of glioblastoma research, a study by Jian et al. reveals critical interactions within the tumor microenvironment that could be targeted to impede angiogenesis, a hallmark of cancer progression.

The Research Topic also features studies that explore the interplay between botanical drugs and the gut microbiota. A research article by Nie et al. and commentary by Yu et al. provides fascinating insights into the therapeutic potential of botanical formulations in influencing the gut microenvironment to augment anti-cancer immune responses. Furthermore, a comprehensive review of Tubeimoside-1 by Wang et al. offers a comprehensive assessment of the therapeutic potential of tubeimoside-1, highlighting its diverse anti-cancer effects. In addition, network pharmacology and experimental validation are employed to investigate the effects and mechanisms of botanical drugs in treating colorectal cancer (Dong et al.; Janani et al.; Shao et al.; Xiang et al.).

As we embrace the era of personalized medicine, the potential of botanical drugs in targeting the tumor microenvironment becomes increasingly evident. From understanding gene signatures to unraveling the interplay with the gut microbiota, these studies illuminate the path toward precision therapies tailored to individual patients. The collective efforts of these research endeavors demonstrate the integration of botanical drugs into the fight against cancer is not only advancing our understanding but also instilling hope for improved outcomes and enhanced patient care.

In conclusion, “*Anti-cancer Effects of Botanical Drugs Targeting the Tumor Microenvironment*” represents a significant step forward in the quest to combat cancer. As we continue to explore the untapped potential of botanical drugs, we embrace the transformative power of the tumor microenvironment in shaping the future of cancer treatment.

